# Determinants of re-operation following tibia intramedullary nailing at a tertiary hospital in south-west Nigeria

**DOI:** 10.11604/pamj.2016.25.250.8642

**Published:** 2016-12-21

**Authors:** Oluwadare Esan, Adetunji Mapaderun Toluse, Oludare Uriel Ashaolu, Ayodele Elkanah Orimolade

**Affiliations:** 1 Department of Orthopaedic Surgery and Traumatology, Obafemi Awolowo University/Teaching Hospitals Complex, Ile Ife, Nigeria; 2 National Orthopaedic Hospital, Igbobi, Lagos, Nigeria

**Keywords:** Reoperation rate, tibia fractures, intramedullary nailing

## Abstract

**Introduction:**

intramedullary nailing is a method of choice in the management of long bone diaphyseal fractures. However, complications necessitating re-operation may arise. This study was aimed at determining the rate and indications for re-operation following intramedullary nailing of tibia shaft fractures.

**Methods:**

it was a cross-sectional study done at Orthopaedic Department of Obafemi Awolowo University Teaching Hospitals Complex, Ile-Ife in Southwest Nigeria. Records of patients who had interlocking nailing for tibia shaft fracture between 2005 and March 2013 were retrieved. Variables of interest extracted included aetiology of fracture, type of fracture, cadre of surgeon and indication for re-operation. Frequency distribution and chi-square analysis were done using SPSS version 22. Level of statistical significance was determined at p-value <0.05

**Results:**

One hundred and forty-six patients had tibia nailing done during the study period. Eighty-six patients met the study criteria with male to female ratio of 2.6:1. There were 51 (59.3%) with open fractures and 35 (40.7%) with closed fractures. Ten patients had re-operation giving a re-operation rate of 11.6 %. Two most common indications for re-operation included loose screw 3 (25%) and surgical site infection (SSI) 3 (25%). There was no statistically significant association between rate of re-operation and the cadre of surgeon (p=0.741) and type of fracture whether closed or open (p=0.190).

**Conclusion:**

Re-operation following tibia intramedullary nailing is an ever present risk. Precautions should be taken to prevent the common indications such as loose screw and surgical site infections.

## Introduction

Tibia is often involved in fractures, and it is predisposed to open fracture because its subcutaneous anteromedial border in its entire length. Some are managed non-operatively especially the closed types [1], while the rest are treated operatively. They are expected to unite within a year following treatment. However, not all patients who had operative treatment will proceed to uneventful union. Re-operation may be necessary in some as a result of complications such as hardware failure, infection and delayed or nonunion [2]. Interlocking intramedullary nailing is the modality of choice because it affords optimal stability of the bone and prepares patient for early rehabilitation [3, 4]. Techniques of inserting the nail and interlocking screws exist and it could either be by closed or open system depending on expertise and the type of system which is available at the facility. The open system involves the use of external jig to place the interlocking screws in the appropriate holes. The closed system of nailing involves the use of image intensifier which is not commonly available in some of the resource poor countries. Whatever technique of fixation employed, surgery may be associated with complications which may occur in the immediate or later on. These complications may necessitate re-operation which will further increase the overall cost of treatment and increase the frequency of hospitalisation of such patients The objectives of the study were to determine the rate and indications for re-operation following intramedullary nailing of tibia shaft fractures as seen in our practice. The knowledge of these indications may help surgeons institute possible preventive measures.

## Methods

This was a cross-sectional study done at Orthopaedic department of a tertiary health facility southwest of Nigeria. The nail used was the solid core stainless steel from Surgical Implant Generation Network (SIGN), USA. Method of reduction was by either open or closed system, without the use of image intensifier. All fractures were classified based on the Arbeitsgemeinschaft fur Osteosynthesefragen/Association for the study of internal fixation (AO/ASIF) grading system. Open fractures were graded using the Gustillo and Anderson system. Re-operation was defined as any surgical procedure done to achieve union, or treat complication of implanted nail and screw within the first year of surgery. Records of skeletally mature patients (18 years and above) who had interlocking nailing for tibia shaft fracture between September 2005 and March 2013 were retrieved. Patients who were followed up for a minimum of one year following fracture fixation for open or closed diaphyseal tibia fractures were included in the study, while those whose follow up did not last up to a year were excluded from the study. Other exclusion criteria were patients with non diaphyseal fractures, patients with diaphyseal fractures with extension into the knee or ankle, and patients that were planned for repeat operation from the time of the initial surgery. Variables of interest extracted included aetiology of fracture, type of fracture, cadre of surgeon and indication for re-operation. The Helsinki protocol was adhered to in the course of the study. Frequency distribution and chi-square analysis were done using SPSS version 22. Level of statistical significance was determined at p-value <0.05.

## Results

**Figure 1:**
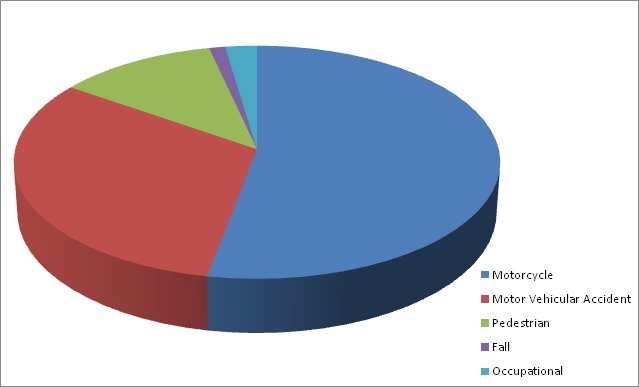
Aetiology of fracture

**Figure 2:**
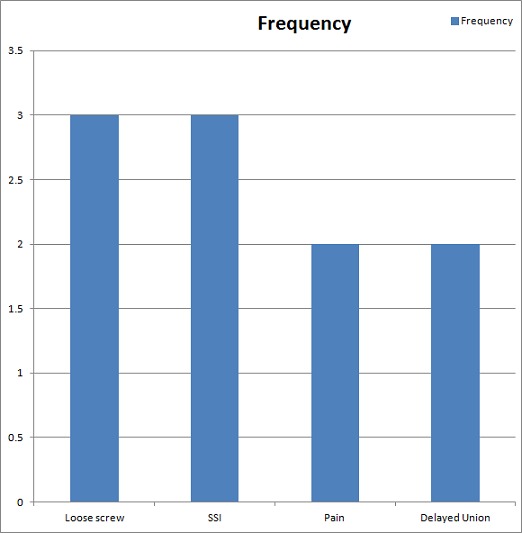
Indications for re-operation

A total of 86 out of 146 patients operated during the period under review for tibia fractures met the inclusion criteria. Male to female ratio was 2.6:1, There were 51(59.3%) cases of open fractures, while closed fractures were seen in 35(40.7%). Motorcycle accident, 45 (52.3%) was the commonest aetiology of fracture Figure 1. Ten patients had re-operation giving the rate of 11.6%. Eight patients out of the 12 that had re-operation had open fracture thereby accounting for about 80% of cases of re-operation Table 1. Most of our patients had fracture classification of A2 and A3 accounting for about 59.3% of the fracture. Two patients fracture pattern could not be classified because the radiographs were not entered in the radiographic database Table 2. Two most common indications for re-operation included loose screw 3 (30%) and surgical site infection (SSI) 3 (30%). Patients with missing radiographs had no revision done and they healed uneventfully from the hospitals records Figure 2. A total of 4 patients had re-operation out of 41 cases done by specialist while 6 cases had re-operation out of 45 cases done by the senior residents. There was no significant association between the rate of re-operation and grade of surgeon (p=0.741). Eight patients (15.7%) had re-operation out of 51 patients who were operated for open fractures while 2(5.7%) had re-operation out of 35 patients who had surgery for closed fractures. This difference was not significant (p=0.190). Two patients who had delayed union had their fractures reduced by open method and their immediate post operative radiographs showed good cortical contacts in 2 views respectively. These 2 patients had their fixations dynamised following which they both proceeded to union. Procedures done for other post-operative complications requiring revision are as shown in the Table 3 below.

**Table 1:** Fracture classification based on AO/ASIF

Fracture Classification	Frequency	Percentage
A1	9	10.5
A2	23	26.7
A3	28	32.6
B1	8	9.3
B2	3	3.5
B3	1	1.2
C1	1	1.2
C2	6	7.0
C3	5	5.8
Missing	2	2.3
Total	86	100

**Table 2:** Revision surgery done per fracture classification

Fracture Classification	Total	Had Revision (%)
A1	9	2(22.2)
A2	23	3(13.0)
A3	28	3(10.7)
B1	8	2(25.0)
B2	3	0(0.0)
B3	1	0(0.0)
C1	1	0(0.0)
C2	6	1(16.7)
C3	5	1(20.0)
Missing	2	0(0.0)
Total	86	12(14.0)

**Table 3:** Revision procedures done following intramedullary nailing

Post –operative indication for revision	Number (n)	Revision procedure done
Surgical site infection	3	Debridement and antibiotics
Loose interlocking screw	3	Removal of loose screws
Delayed union	2	Dynamisation (static interlocking screw removal)
Chronic pain over interlocking screw	2	Removal of irritating interlocking screw

## Discussion

Re-operation is an undesirable event following any surgical intervention. It increases the overall cost of treatment and at the same time prolong patient's hospital stay. However, this undesirable situation does occur. Hence, there is the need to identify the frequency of re-operation and identify the indications so as to mitigate the factors as much as possible. The overall rate of re-operation in this study was 11.6%. Most of the literatures on re-operation are focused on single indications such as nonunion or infection and as such data on overall rate of re-operation following tibia nailing seems to be scanty. Bhandari et al found overall re-operation rate of 16.9% in patients that had reamed nailing and 18.9% in patients that had unreamed nailing of the tibia within one year [5]. Bhandari et al in a meta analysis further found that the annual re-operation rate following intramedullary nailing of tibia fractures ranges between 12-44% [3]. Open fractures and other factors were identified to be associated with more re-operation in their series. Findings from our study show that our re-operation rate is comparable with results of other workers. In our series, all the patients had reamed intramedullary nailing for both open and closed tibia fractures. Reamed intramedullary nailing is a preferred choice because of reported advantages of providing optimal mechanical stability, rapid fracture union and low incidence of secondary procedure [6–10]. Some authors have found that the rate of nonunion following open tibia fracture that will necessitate re-operation varies from 4%-48% [4]. We identified deep infection and loose screws as the commonest indication for re-operation in our series. Gustilo et al found that Patients with open fractures are expected to have a higher predisposition to deep infection compared with ones with closed fractures [11]. Schemitsch et al also corroborated this observation in their meta-analysis at identifying predictive factors for re-operation following tibia intramedullary nailing [12]. Screw loosening was another complication recorded in our series. These were consequently removed and the fractures united. Gaebler et al identified that loosening is a known complication of interlocking nailing but not a single of their patients required revision [13]. They recognized loosening when partially threaded interlocking screws were used compared with fully threaded screw. In our series, our screws were threaded at the tips and the base. There was radiographic evidence of osteolysis around the screws. These loose screws were removed as minor procedure and this did not affect union in these patients. Loosening may be as a result of thermal injury to the bone while drilling the interlocking hole with high speed drill. Gaebler et al [13] also recorded significant level of screw breakages in patients with open fractures. Broken hardware were not recorded in our study as a complication probably because all our patients had reamed nailing which is said to be associated with less implant failure compared with unreamed [14]. We recorded a rate of 2.3% in delayed union in our series. These patients had no such documented risk factors for delayed union such as open fracture, fracture comminution and other co-morbidities. A further 2 patients had chronic pain at the site of the interlocking screws necessitating the removal of these screws. The screws were distal in location. This could be due to injury to a nerve along the tract of the interlocking screw with formation of a neuroma. The removal of the screw did not yield any immediate relief of pain. Apart from anterior knee pain which is well documented following tibia nailing, not much has been documented/studied about chronic pain along interlocking screw tract. This seems to be an uncommon finding from our study.

## Conclusion

Re-operation following tibia intramedullary nailing is an ever present risk. Adequate measures should be taken to prevent the common indications such as loose screw and surgical site infections.

### What is known about this topic

- That infections and complications of union may accompany fracture fixation with interlocking nails. Most of these cases were following fresh fractures.

### What this study adds

- We interestingly used both open and closed methods of reduction because some of our patients presented after 1 year with non union following fracture management by the traditional bone setters;
- We also found a complication of chronic pain at the interlocking screw scar which we haven’t seen in the literatures reviewed.

## References

[1] Lindsey RW, Blair SR (1996). Closed tibial shaft fractures: which ones benefit from surgical treatment?. J Am Acad Orthop Surg..

[2] Bhandari M, Tornetta P, Sprague S, Najibi S, Petrisor B, Griffith L (2003). Predictors of re-operation following operative management of fractures of the tibial shaft. J Orthop Trauma..

[3] Bhandari M, Guyatt GH, Tong D, Adili A, Shaughnessy SG (2000). Reamed versus nonreamed intramedullary nailing of lower extremity long bone fractures: a systematic overview and meta-analysis. J Orthop Trauma..

[4] Bhandari M, Guyatt GH, Swiontkowski MF, Schemitsch EH (2001). Treatment of open tibia shaft fracture: a systematic overview and metanalysis. J Bone Joint Surg Br..

[5] Bhandari M, Guyatt G, Tornetta P, Schemitsch EH, Swiontkowski M, Sanders D (2008). Randomized Trial of Reamed and Unreamed Intramedullary Nailing of Tibial Shaft Fractures. J Bone Joint Surg Am..

[6] Xia L, Zhou J, Zhang Y, Mei G, Jin D (2014). A meta-analysis of reamed versus unreamed intramedullary nailing for the treatment of closed tibial fractures. Orthopedics..

[7] Larsen LB, Madsen JE, Høiness PR, Øvre S (2004). Should insertion of intramedullary nails for tibial fractures be with or without reaming? A prospective, randomized study with 3.8years’ follow-up. J Orthop Trauma..

[8] Briel M, Sprague S, Heels-Ansdell D (2011). Economic evaluation of reamed versus undreamed intramedullary nailing in patients with closed and open tibial fractures: results from the study to prospectively evaluate reamed intramedullary nails in patients with tibial fractures (SPRINT). Value Health..

[9] Anglen JO, Blue JM (1995). A comparison of reamed and unreamed nailing of the tibia. J Trauma..

[10] Finkemeier CG, Schmidt AH, Kyle RF, Templeman DC, Varecka TF (2000). A prospective, randomized study of intramedullary nails inserted with and without reaming for the treatment of open and closed fractures of the tibial shaft. J Orthop Trauma..

[11] Gustilo RB, Mendoza RM, Williams DN (1984). Problems in the man-agement of type III (severe) open fractures: a new classification of type III open fractures. J Trauma..

[12] Schemitsch EH, Mohit Bhandari, Sander DW, Swiontkwoski M, Tornetta P, Zdero R (2012). Prognostic factors for predicting outcomes after intramedullary nailing of the tibia. J bone Joint Surg Am..

[13] Gaebler C, Berger U, Schandelmaier P, Greitbauer M, Schauwecker HH, Applegate B (2001). Rates and Odds Ratios for Complications in Closed and Open Tibial Fractures Treated With Unreamed, Small Diameter Tibial Nails: A Multicenter Analysis of 467 Cases. J Orthop Trauma..

[14] Duan X, Al-Qwbani M, Zeng Y, Zhang W, Xiang Z (2012). Intramedullary nailing for tibial shaft fractures in adults. Cochrane Database of Systematic Reviews..

